# Ulcer of the Rectum and Anus

**Published:** 1872-12

**Authors:** John E. Owens

**Affiliations:** Surgeon St. Luke’s Hospital; Chicago


ARTICLE V.—*Ulcer of the Rectum and Anus.* By JNO. E. OWENS, M.D., Surgeon St. Luke's Hospital.

A mere description of ulcer of the rectum and anus, at first thought, seems somewhat presumptuous. Meeting, however, yearly, in hospital and private practice, with a certain number of cases, which have been treated by different medical gentlemen with suppositories, ointments, injections and caustics, unsuccessfully one need scarcely say, a few words devoted to the above subject may not be out of place.

Popularly, the term "piles" is used to designate almost every disease of the anus and rectum, and pain there located. Being too frequently diagnosed "neuralgia," the existence of an irritable ulcer is overlooked, for want of a thorough examination. I say *thorough* examination, because the ulcer, being occasionally no larger than a large pin head, or small pea, the case may escape a correct diagnosis by anything short of a very careful exploration of the part. One accustomed to look for and treat this affection, must frequently be amazed, no less by the fact that such intolerable suffering should originate in a disease so insignificant in extent, than by that of the prompt relief from pain, and the rapid cure effected by a proper performance of the requisite operation. This variety of ulcer is situated partly within the rectum and partly within the anus.

In order to make a satisfactory examination, the bowels having been previously well evacuated, the patient must be anæsthetized, and placed in a good light. Under these circumstances, with the use of Sim's speculum, the rectum may be explored as far as is necessary. Having obtained the patient's consent to any necessary operative procedure, the examination and operation may be made at one ætherization. There is no protrusion at the anus, in the disease in question, unless as a complication; but there is, in all cases, a slight purulent discharge, and occasionally a few drops of blood trickle away. The quantity of the latter is much less than that accompanying hæmorrhagic piles. Pain in passing the contents of the bowels, is the prominent symptom. This is peculiar and striking, and instead of diminishing, at the cessation of the act, increases in severity, and continues for a period varying from some minutes to five or six hours, and perhaps longer. In uncomplicated cases it then ceases, and there is no suffering until the bowels are again moved, when there is a repetition of the suffering. The symptoms are progressive, and Nature, unaided, under the circumstances of the location of the ulcer, is powerless to effect a cure.

As far as my experience goes, the ulcer is situated on one side, far back towards the posterior segment of the anus. In a large proportion of cases of ulcer of the anus and rectum, a small excrescence is visible externally, and the ulcer is seen situated at the base of this little tumor, which, in consequence, becomes a sign of great importance. The disease originates in a variety of ways. In a large majority of cases, some breach of surface is produced by straining, and in consequence of the periodical disturbance of the part, the ulceration increases, and assumes a definite form. In other cases, the disease originates in a pile situated at the verge of the anus. The pile, in consequence of pressure and friction, inflames, becomes excoriated, and finally ulcerates. The ulcerative process extends into the rectum by continuity of tissue. In other cases again, the disease arises from the abscess that results in anal fistule, or spreads from the rectal orifice of the fistule. In the latter class, the ulcer begins where the fistule ends, or, in other words, it radiates from the internal orifice of the fistule, and bears the same relation to the fistule that a fan does to its handle. In such cases, if the fistule is superficial, an operation must be performed, not only for the cure of the fistule, but likewise for that of the ulcer, as in the following case:

Case 1. A gentleman, aged fifty-one years, had suffered from hæmorrhoids since 1849. During the same year, an anal fistule supervened, through which, at times, blood squirted, whilst the patient was at stool. In 1861 he received some palliative medical treatment for hæmorrhoids. During the present year, the gentleman consulted me on account of an agonizing pain located at the anus. He had been for years a great sufferer. The pain commenced whilst at stool, and grew so severe after the act of defecation, that frequently the patient was obliged to take his bed for several hours. In consequence of this severe periodical distress, the patient was often obliged, from sheer prostration, to disregard engagements, both of business and pleasure. The anus was constantly moistened with a discharge more or less purulent. The character of the pain indicated ulcer of the anus and rectum. The bowels having been evacuated, the patient ætherized and placed in a good light, the fistule was at once discovered, but a cluster of hæmorrhoids in a great degree obscured any further view. The piles were at once ligated, and the fistule laid open upon a grooved director. This seemed sufficient for the time. Between two and three weeks later, after the piles had sloughed off, and the inflammatory swelling in a considerable degree subsided, the patient was again ætherized, and an ulcer of the size of a dime was discovered at the site of the inner orifice of the fistule. An incision, commencing a little above the ulcer, was carried through it and a portion of the fistulous track, dividing at the same time the greater part of the sphincter ani muscle. The pain, which was as severe, and as characteristic of ulcer of the rectum and anus, subsequent to the first operation as before it, now at once subsided, and the patient found himself, in feeling, a new man. After the first operation, morphia, in half and three-quarter grain doses, was administered, for the relief of pain. After the last operation, morphia was only used to lock up the bowels. Fistules, complicated with ulcer of the anus and rectum, heal very slowly, and sometimes not at all, unless the underlying muscular fibres of the sphincter ani muscle are incised. At the time of the last operation, two polypoid growths were removed from the rectum. These latter originated, probably, in hæmorrhoids, and acquired their peculiar shape from pressure of the sphincter muscle, which becomes highly developed in cases of ulcer. At every stool these growths were extended, and it was always with more or less difficulty that they were replaced.

The following are typical cases of ulcer of the anus and rectum:

Case 2. A gentleman, near the middle age of life, consulted me for what he imagined to be piles. A few drops of blood occasionally trickled from the anus, which was usually moistened by a purulent discharge. He had for months felt more or less distress at the anus, but, following every act of defecation, came the characteristic gnawing and paroxysmal pain, which made life a burden. The patient having been ætherized, a somewhat excavated and irregular ulcer, about the size of a silver quarter, was discovered. The ulcer commenced at the muco-cutaneous margin of the anus, and extended into the rectum. A free incision was made through the ulcer and sphincter ani muscle. The patient's sufferings ceased on the day of the operation, and there has been no return of them. In forty-eight hours he was able to leave his bed, and to attend, in a certain degree, to his business.

Case 3. A young married lady had suffered from constipation during pregnancy. Whilst in that condition, and after the birth of her child, defecation was accomplished by means of considerable voluntary tenesmus. There was a scanty discharge of pus, and occasionally a little blood. The act of defecation, attended with some smarting and burning, was followed by great distress, located at the anus. This lady, for the most part able to attend to her household duties, was prostrated with pain for several hours after going to stool. The ulcer, together with the underlying muscular fibres, was incised. The characteristic pain was no longer felt, and the patient made a rapid recovery.

Case 4. A young lady, in the summer of 1871, consulted me for a most intolerable pain of the rectum. She had suffered agonies for several months. Pain of a moderate degree was in this case continuous, but following the act of defecation, after a very short interval, an almost unbearable pain, located at the anus, seized the patient, and continued for several hours, after which it subsided in a certain degree, to return at the next act of defecation. An angry-looking, irregular ulcer, together with the muscular fibres of the sphincter, was incised, and the ulcerated and cut surfaces freely cauterized. The operation was followed by agonizing pain, that could only be subdued by chloroform. I attributed this to the cauterization of a very angry-looking sore. The patient made a good but rather tardy recovery.

Sim's speculum was used in each case. Cauterization, as an adjunct, is unnecessary in most if not all cases. The ulcer is situated on the muscular fibres of the sphincter ani muscle. These muscular fasciculi are thrown into an unnatural contractility during, but especially immediately following, every act of defecation; hence the severe paroxysmal pain by which the affection is characterized. For the cure of this disease, the French surgeon, Boyer, and those who imitated him, were induced to make a complete division of the sphincter ani. This operation succeeded in effecting a cure, but it was reserved for an English surgeon (Copeland) to show that a simple incision through the ulcer, commencing a little above it, and including the superficial layers of muscular fibres underlying it, is sufficient. The operation promptly arrests the pain, by cutting short the undue muscular contractility of the muscular fibres lying at the bottom of the ulcer. The production of an atonic state of the sphincter, by forcible dilatation, has been recommended, but the operation is now considered unsurgical. In several cases I have both incised and used forcible distension, but the former alone is sufficient. The after-treatment is simple, viz.: keep the bowels closed for four or five days, bathe the part for the sake of cleanliness, and, finally, use some laxative, to a moderate extent, in order that the contents of the bowels may do no violence to the wounded bowel and anus.

